# The interplay between teachers’ value-related educational goals and their value-related school climate over time

**DOI:** 10.1007/s10212-024-00849-y

**Published:** 2024-06-17

**Authors:** Thomas P. Oeschger, Elena Makarova, Evren Raman, Beatrice Hayes, Anna K. Döring

**Affiliations:** 1 Institute for Educational Sciences, University of Basel, Basel, Switzerland; 2 School of Social Sciences, University of Westminster, London, UK; 3 Department of Psychology, Royal Holloway University of London, Egham, UK

**Keywords:** Values, Value formation, Value transmission, School climate, Primary school

## Abstract

Values education within the school context is, among other elements, shaped by a value-related school climate as well as teachers’ value-related educational goals. This longitudinal study investigated the interplay between these two elements over fifteen months, starting in March 2021, and including four points of measurement (*t1* − *t4*). The sample consisted of 118 primary school teachers (years 1 and 2) from primary schools in Switzerland. Teachers’ value-related educational goals were measured with the *Portrait Values Questionnaire*, and teachers’ perception of their school climate was measured with the *12-Item School Climate Measure Scale*. *Random Intercept Cross-Lagged Panel Models* along with *Multiple Imputation* for missing data were used to investigate the reciprocal relationships along the four dimensions of value-related educational goals represented by Schwartz’s *Higher-Order Value Types: Openness to Change*, *Conservation*, *Self-Enhancement*, and *Self-Transcendence* and their corresponding dimensions of a perceived value-related school climate of *Innovation*, *Stability*, *Performance*, and *Support.* For the dimensions “*Innovation* and* Openness to Change*,” the analyses revealed that the perceived value-related school climate of *Innovation* predicted teachers’ value-related educational goals of *Openness to Change* significantly from *t1* to *t2*, while an effect in the opposite direction from *t2* to *t3* and from *t3* to *t4* was found. For the dimension “*Stability* and *Conservation*,” the analyses revealed that the perceived value-related school climate of *Stability* predicted teachers’ value-related educational goals of *Conservation* from *t3* to *t4*. These findings are discussed in light of the dynamic processes of values education within the school environment as well as in the context of environmental and societal developments.

**Supplementary Information:**

The online version contains supplementary material available at 10.1007/s10212-024-00849-y.

## Introduction

The transmission of values as a process of passing behaviors and attitudes on to the next generation (Schönpflug, 2001) is considered one of the central tasks of a society (Roest et al., 2010; Rohan & Zanna, 1996). The family environment can thus be seen as the primary instance of socialization in a society (Tillmann, 2020). As the main instance of secondary socialization (Tillmann, 2020), schools are of major importance in transmitting values, and their relevance in this context is increasingly being discussed internationally (Matthes, 2004). Schools play a key role in the development of personal values orientations in different contexts (Beck, 1990; Halstead, 1996). Fend (2008) identifies four social functions of schools and highlights the importance of their *integration* and *cultural transmission function* for ensuring, as an institution, the integration of children and adolescents into society through the transmission of values and norms that underlie the democratic and constitutional order.

Major aspects of these values-transmitting processes in schools are the interactive processes that aim to develop, strengthen, or change the value-related behavior of children by means of intended actions by teachers through values education. Values education refers to the conscious and intended teaching of social, political, cultural, and aesthetic values (Veugelers and Vedder, 2003), which are an integral part of educational policy guidelines, curricula (Oeschger et al., 2022), teaching materials, educational laws, and school mission statements (Welten, 2022), as well as of the prevailing school climate (Berson & Oreg, 2016) and the daily classroom management styles of teachers (Barni et al., 2018).

Two specific elements involved in values education in schools—the school climate and teachers with their value-related educational goals—are of particular interest. On the one hand, a school climate that is shaped by the values that prevail in it contributes directly to the development of children’s and young people’s personal values orientations, such as the prosocial behavior of children (Villardón-Gallego et al., 2018) and adolescents (Luengo Kanacri et al., 2017; Barr and Higgins-D'Alessandro, 2007). On the other hand, teachers play an explicit role in values education in schools as they are important cultural bearers, who tend to be close to a broad consensus of values in society (Schwartz, 1992). Teachers therefore convey social, common values, and norms through their daily interactions in the classroom via their classroom management (Cadima et al., 2015) and with their value-related educational and socialization goals (Tamm et al., 2020).

While several studies have considered the processes of value transmission in the school environment (e.g., Luengo Kanacri et al., 2017; Berson & Oreg, 2016; Daniel et al., 2013), there is little empirical evidence on how certain elements involved in values education in schools interact with each other. Even fewer studies have considered this relationship over time.

The present study aims to fill this gap in the research by empirically analyzing for the first time how teachers’ value-related educational goals and their perceived value-related school climate interact with each other over time. The study of this interaction over time will make a valuable and novel contribution to the discussion of exchange mechanisms in the context of values transmission and values education in schools.

### Schwartz’s theory of basic human values

Values are at the heart of society and are used to describe cultural groups, or individuals. They characterize motivational bases of behavior and attitudes, and are considered general goals (e.g., helpfulness) that guide an individual in life (Bardi & Schwartz, 2003; Maio, 2010). *The Theory of Basic Human Values* (Schwartz, 1992, 1994) summarizes the most important features of the structure of basic human values. Considered the most widely recognized theory to date, it has since been validated in more than 80 countries, taking into account different geographic, cultural, linguistic, religious, age, gender, and occupational groups in each case (Bilsky et al., 2010; Davidov et al., 2008; Schwartz & Rubel, 2005). The theory provides a sound basis for reliable empirical research methods on values and for this reason forms the theoretical framework of this study.

**Fig. 1:**
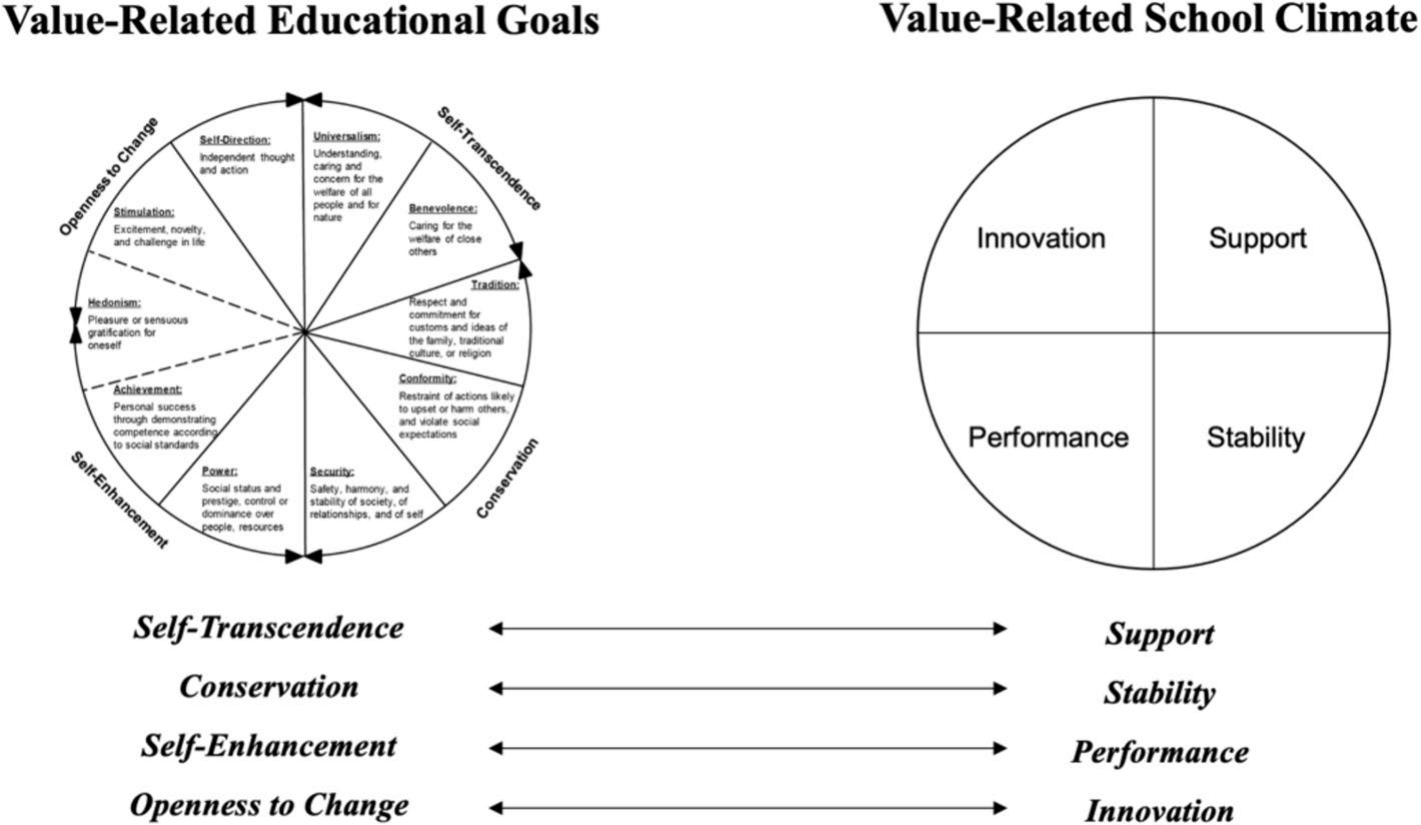
Analyzed dimensions of teachers’ value-related educational goals and teacher’s perception of their value-related school climate according to *Schwartz’s Theory of Basic Human Values*, own illustration

Organizing his framework in a circular structure Schwartz subsumes values under the heading of ten *Basic Value Types* such as *Universalism*, *Benevolence*, *Tradition*, *Conformity*, *Security*, *Power*, *Achievement*, *Hedonism*, *Stimulation*, and *Self-Direction*, which define their central motivational goals (Schwartz, 1994). Considering the motivational compatibility of values, the model further describes two dimensions of opposite poles (*Higher-Order Value Types*). The first dimension indicates the conflict between *Self-Enhancement* values (with their focus on reaching personal goals and controlling others) and *Self-Transcendence* values (which focus on the well-being and interests of others). The second dimension portrays the conflict between *Openness to Change* values (with their focus on change and excitement) and *Conservation* values (which focus on stability and maintaining the status quo) (see Fig. 1, left side).^1^

**Fig. 2:**
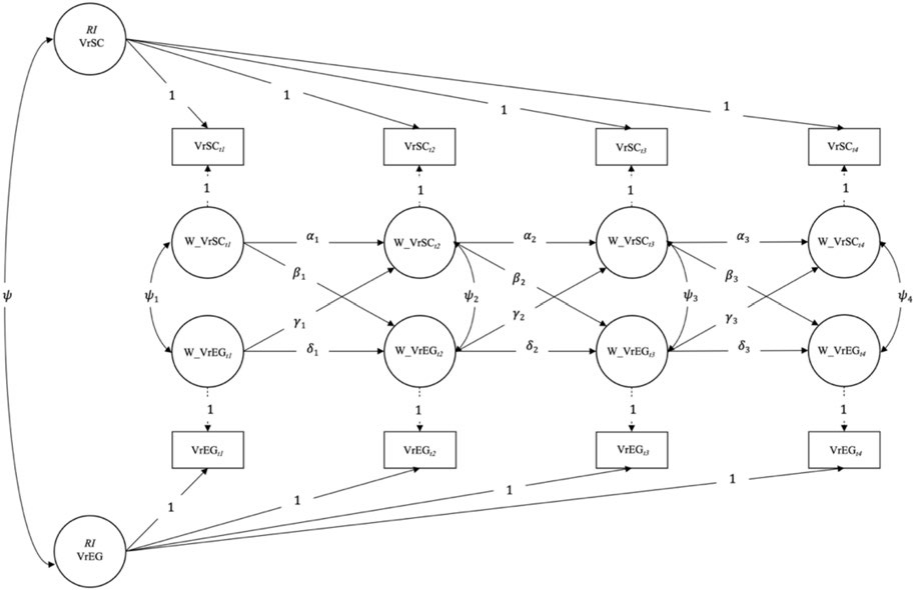
General illustration of a Random Intercept Cross-Lagged Panel Model (RI-CLPM) for teacher's perceived value-related school climate (VrSC) and teacher’s value-related educational goals (VrEG). Note. The following parameters are estimated in the Random Intercept Crossed-Lagged Panel Model (RI-CLPM) of teachers’ value-related educational goals (VrEG) and their perceived value-related school climate (VrSC) over four time points t1-t4: (1) between-level correlation ($\psi$) between the two random intercepts (RI) of teachers’ perceived VrSC (RI VrSC) and teachers’ VrEG (RI VrEG); (2) within-level correlations between the residual components of VrSC (W_VrSC) and VrEG (W_VrEG) for every specific timepoint t1-t4 ($\psi$ 1-$\psi$ 4); (3) within-level (W) carry-over effects for VrSC (W_VrSC) (α1-α3) and VrEG (W_VrEG) (δ1-δ3) for every wave (t1-t2, t2-t3, and t3-t4); and (4) within-level (W) spill-over effects from VrSC (W_VrSC) to VrEG (W_VrEG) (β1-β3) and from VrEG (W_VrEG) to VrSC (W_VrSC) (γ1-γ3) for every wave (t1-t2, t2-t3, and t3-t4)

### Values education in schools and teachers’ value-related educational goals (VrEGs)

Values education consists of all educational efforts made towards nurturing and developing the awareness of positive values in children and towards advancement in line with their own potential (UNESCO, 2020). In schools, values education refers to the interactive methods used by teachers to intentionally develop, reinforce, or modify the values-based behavior of students.

Teachers’ role as values educators is therefore increasingly emphasized (Sutrop, 2015; Thornberg, 2008; Veugelers, 2000). Teachers convey common social values and norms through their daily classroom interactions with their students by way of communicating expectations for participation and problem solving, modelling attitudes, classroom management practices, structuring of learning environments and encouragement through rewards (Wentzel & Looney, 2007). During intended interpersonal processes aimed at teaching values, they promote the development of students’ values by acting as role-models, establishing shared social spaces in the classroom, providing opportunities for decision-making, or encouraging cooperation (Colnerud, 2006; Halstead & Taylor, 2000).

These processes of values education in terms of teaching are, among other things, shaped by teachers’ value-related educational goals, which are constituted on norms and values prevailing and accepted in society (Veugelers and Vedder, 2003). Teachers’ value-related educational goals are considered a key element in values education in the school environment; they are the basis for intended interactive processes of values education that aim to develop, reinforce, or change children’s value-related behaviors through intentional actions (Standop, 2013).

### Value-related school climate (VrSC)

A school climate can be seen as a multicomponent construct that reflects norms, goals, interpersonal relationships, teaching and learning practices, and organizational structures grounded in patterns of students’ and teachers’ shared experiences around school life (National School Climate Council, 2007). The school climate has a bidirectional relationship with school stakeholders (Altuntaş et al., 2020). As we see in organizations more generally (Arieli et al., 2018), a school climate consists of unwritten beliefs, values, and attitudes that involve direct interactions between involved stakeholders (Korkmaz, 2008; Welsh, 2000). When it comes to values education, there is a common sense that the school climate—as a system of shared attitudes, beliefs, and values (Haynes et al., 1997)—plays an important role in the development and transmission of values and norms (Higgins-d’Alessandro & Sadh, 1997; Lang et al., 1999). With reference to Berson and Oreg (2016), such value-related school climates can thus correspond to four value dimensions, namely, the degree to which the school climate reflects an emphasis on *Stability* (corresponding to *Conservation* values like *Security* or *Tradition*), *Support* (corresponding to *Self-Transcendence* values like *Benevolence* or *Universalism*), *Innovation* (corresponding to *Openness-to-Change* values like *Stimulation* or *Self-Direction*), and *Performance* (corresponding to *Self-Enhancement* values like *Achievement* or *Power*).

Teachers both actively shape their school climate and are shaped by it. This mutual influence is shown in several studies and refers to various aspects such as *Commitment* (Kahn, 2019), *Leadership* (Kilinç, 2014), *Achievement Goal Orientation* (Dickhäuser et al., 2021) *Innovation* (Ozdemir & Cakalci, 2022) or *Collaboration* (Öngel & Tabancalı, 2022). Lovat and Clement (2008) found that values education can transform an educational environment completely by forming an inclusive school ethos, and Lovat et al. (2009) emphasize the positive effect of values education on the atmosphere in a school environment.

Based on the evidence for the mutual influence of teachers and their school climate on the various aspects, it can be assumed that in the context of values education in the school environment, teachers’ value-related educational goals and their value-related school climate might also influence each other. To date, the authors are not aware of any study that examines this bidirectional influence. We assume that interactions between teachers’ value-related educational goals and their perceived value-related school climate (*Stability* and *Conservation*, *Support* and *Self-Transcendence*, *Performance* and *Self-Enhancement*, and *Innovation* and *Openness to Change*) exist, and in conducting this study we offer a novel approach to empirically analyzing teachers’ value-related educational goals and their perceived value-related school climate over time, applying Schwartz’s *Theory of Basic Human Values*.

### The present study

The impact of the role of teachers with their value-related educational goals as well as the impact of a value-related school climate in children’s values education can be taken as given, on the basis of numerous studies (see the previous section). The authors were interested in what the reciprocal relationships between these two elements might be. Therefore, this study aims to answer the following research questions, all the while applying Schwartz’s theoretical framework of the *Theory of Basic Human Values*:

1. How do teachers’ value-related educational goals and their perceived value-related school climate *predict* themselves over time (*carry-over effects*)?
2. How do teachers’ value-related educational goals and their perceived value-related school climate *predict* each other over time (*spill-over effects*)?

Correlative and regressive procedures (path analyses) were used to analyze how the teachers’ value-related educational goals and their perceived value-related school climate can be predicted across each of four measurement time points and how they are related to themselves (question 1). The reciprocal interaction of the teachers’ value-related educational goals on their perceived value-related school climate across the four measurement time points and vice versa (question 2) was examined using a *Random Intercept Cross-Lagged Panel Model* (RI-CLPM; Hamaker et al., 2015). Schwartz’s *Theory of Basic Human Values* lends itself very well to this study in that the scales used in comparing the two constructs are the same (dimensions of *Higher-Order Value Types*) and can thus be well compared statistically (see Fig. 1).

## Method

This study is embedded in an ongoing larger longitudinal international research project on the formation of children's values in schools. The project will highlight how primary schools shape children’s personal value development by employing a longitudinal design in Switzerland along with a comparative cross-sectional study in the UK.^2^

### Participants and procedures

The sample in this study included 118 primary school teachers (108 females, 10 males) recruited with the permission of their respective education departments from public primary schools in Switzerland. The teachers all taught grade 1 at the beginning of this study (time point *t1*)*.* The age of the sample ranged from 21 to 64 years with a *M*_age*t1*_ = 38.33 (SD = 13.04). In all, 104 teachers (91.5%) were born in Switzerland with 14 (8.5%) born in another country. Their average teaching experience at *t1* was 12.8 years (range 1–39 years, SD = 11.2). In all, the survey ranged over two school years (from August 2020 to August 2022), i.e., from the start of year 1 to the end of year 2 (the two first years of primary school in Switzerland). The participating teachers provided data on their value-related educational goals and their perceived value-related school climate at four time points (starting in March 2021) over a 15-month period with lags of approximately 3 months. Over the four time points, all participants stayed in the same school environment and completed the questionnaire as follows: *t1*: (March–May 2021); *N* = 108, *t2*: (August–October 2021); *N* = 102, *t3*: (January–February 2022); *N* = 96, *t4*: (May–June 2022); *N* = 84. Complete data across all four time points (*t1-t4*) are available from *N* = 76 teachers. As a result of the pandemic situation in Switzerland (five pandemic waves starting between March 2020 and February 2022) (Oxenius & Karrer, 2022), special measures (distance and hygiene rules, mask requirement, and testing) were in place in schools at time point *t1* (pandemic waves two and three) and timepoint *t2* (pandemic wave four). Time points *t3* (pandemic wave five) and t4 (no more pandemic situation) were not affected by the measures. At the end of February 2022, the Russian invasion of the Ukraine began in full force, and European societies in general, and schools in particular, faced the challenge of having to accommodate for the learning of refugee children from the Ukraine at very short notice.

 Unipark’s EFS Survey tool (version 22.2) was used for the online surveys, and the individual survey links were emailed to the teachers 1 week before the survey was launched. This study was approved by the ethical committee of the University of Basel. All data collected were anonymized, and thus, no conclusions could be drawn about the corresponding individuals.

Due to the possible attrition of teachers (absence, illness at a single time point) within and across time, the missingness in this dataset were evaluated using Little’s MCAR test, which returned a non-significant chi-square statistic (*χ*^2^ = 637.027, df = 671, *p* = 0.823), indicating that the data are missing completely at random (MCAR). This finding confirms that the statistical assumptions for the subsequent imputation procedure (see below) and the statistical analysis have been met.

### Measures

#### Value-related educational goals

The *Portrait Values Questionnaire* (PVQ-21, Schwartz, 1994) was used to assess teachers’ value-related educational goals. The *PVQ-21* consists of 21 items that include a brief verbal portrait describing a person’s life goals or aspirations. This survey was not focused upon teachers’ personal values, but on their value-related educational goals. These are defined as the values that teachers want to promote in their students. This operationalization was already applied and validated among parents (Barni et al., 2018; Döring et al., 2017; Tamm et al., 2020).

Participants were presented with the following brief: “Imagine that the pupils in your class were to fill in this questionnaire. How would you like your pupils to complete it? It is not about what the children are really like, but about what answers you would like them to give. How similar do you want your pupils to be to the people described?”.

Using a 6-point Likert scale (from 1 = “not at all like them” to 6 = “very much like them”), teachers rated how much they wanted their students to resemble the person described in each of the 21 portraits. This determines how similar they would like their students to be to the 21 value-related person descriptions. Several items each represent the 10 *Basic Value Types* defined by Schwartz. For example: “They believe that people should do what they’re told. They think people should always follow rules, even when no-one is watching” or “Tradition is important to them. They try to follow the customs handed down by their religion or their family” represents the *Basic Value Type* of *Tradition*. The 21 original statements were translated into German for this purpose. The analysis focused on the four *Higher-Order Values* (*Conservation*, *Openness to Change*, *Self-Enhancement*, and *Self-Transcendence*) because they allow us to compare them theoretically to the four dimensions of the perceived value-related school climate (*Support*, *Stability*, *Performance*, and *Innovation*).

In addition, Cronbach’s alpha scores were calculated for the multiple scales across the five multiply imputed datasets. Across all datasets, the scales showed generally high reliability, with alpha scores ranging from 0.54 to 0.94. The mean (and range) alpha scores for each scale across all five datasets were as follows: *Conservation* (0.85), *Self-Transcendence* (0.94), *Self-Enhancement* (0.54), and *Openness to Change* (0.91). These results suggest that all scales showed satisfactory to excellent reliability except for the *Self-Enhancement* scale.

#### Value-related school climate

To analyze the teachers’ perceived value-related school climate the *12-Item School Climate Measure Scale* (Berson & Oreg, 2016) was used, which was originally adapted from the *Organizational Culture Profile (OCP) instrument* of O’Reilly et al. (1991). The *12-Item School Climate Measure Scale* consists of 12 items dealing with four dimensions of school climate (*Supportive*, *Innovative*, *Performance*, and *Stability*). Participants were presented with the following brief: “The following questions relate to the climate in your school. For each statement, please tick the box that applies to you. At my school …?”.

Using a 5-point Likert scale (from 1 = “not at all” to 5 = “a lot”) teachers rated the school climate at their school in response to each item. For example: “People at my school help one another” or “There is a supportive atmosphere at my school” represents the value-related school climate dimension *Supportive*. The 12 original statements were translated into German for this purpose and were purpose-adapted with the phrase “at my school”.

Additionally, Cronbach's alpha scores were calculated for the multiple scales across the five multiply imputed datasets. Across all datasets, the scales showed generally high reliability, with alpha scores ranging from 0.81 to 0.91. The mean alpha scores for each scale across all five datasets were as follows: *Supportive* (0.91), *Stability* (0.81), *Performance* (0.86), and *Innovation* (0.89). Overall, the four subscales demonstrated good to excellent reliability across five multiple imputed datasets, as evidenced by their mean Cronbach’s alpha scores.

### Data analytic approach

#### Centering

The rating scores of the 21 value items from the value-related educational goals and of the 12 items from the perceived value-related school climate were centered on the respective mean of all items for each participant in order to correct for individual differences in use of the response scale. Centering has become the usual procedure in values research (Bardi et al., 2014). This procedure is also suitable for eliminating multicollinearity between individual data and context characteristics and for reducing covariances between regression coefficients and constants.

#### Aggregating

To reduce the complexity of the models, the mean scores of all item scores belonging to each specific dimension of value-related educational goals (*Openness to Change*, *Self-Enhancement*, *Self-Transcendence*, and *Conservation*) and all item scores of each specific value-related school climate dimension (*Supportive*, *Performance*, *Stability*, and *Innovation*) were computed to deal with observed (manifest) scores for each time point.

#### Random Intercept Cross-Lagged Panel Model

To answer the research question and to analyze the relations between the four specific dimensions of the value-related educational goals and the perceived value-related school climate, the “basic” *Random Intercept Crossed-Lagged Panel Model* (RI-CLPM) (Hamaker et al., 2015) as shown in Fig. 2 was used.

The RI-CLPM has been recently favored over the “classic” *Crossed-Lagged Model* (CLPM) because it breaks down observed scores into dynamic *within*-person and stable *between*-person differences and accounts for stable, trait-like differences *between* persons; that the lagged relations pertain exclusively to *within*-person fluctuations. The within-person effects capture three types of relationships: (a) simultaneous, time-specific correlations at the same time point, (b) *carry-over* (autoregressive) effects from time points *t*_*i*-1_ to *t*_*i*_ and (c) *spill-over* (cross-lagged) effects from time points *t*_*i*-1_ to *t*_*i*_ (see Mulder & Hamaker, 2020). RI-CLPMs were run to analyze the effects between all four theoretically founded dimensions of the value-related educational goals and perceived value-related school climate.

We opted against employing constrained models due to the dynamic, multidimensional and evolving nature of the relationships we were examining between perceptions of the value-related school climate and the value-related educational goals of the teachers. This decision was grounded in both conceptual and methodological considerations. The focus of this exploratory research was on capturing the fluidity and variability inherent in these relationships over time, especially considering the window in which data collection took place. Constrained models are not relevant to the current study as the primary interest lies in understanding how paths vary over time rather than maintaining the assumption of consistency. In addition, the open-ended nature of exploratory research necessitates the use of unconstrained models. These models are better suited to accommodating the nuanced investigation required, ensuring that the methodological approach is coherent with the research objectives (Preacher et al., 2013).

All models for the four dimensions were first estimated with maximum likelihood estimation with standard errors robust to non-normality (MLR) method in R (4.3.0), R Studio (2023.03.1 + 446) and Lavaan (0.6–15). Missing data was handled with the full information maximum likelihood procedure (FIML; Arbuckle, 1996). In addition to *χ*^2^ tests, the following indices were also considered alternative indicators of model fits in evaluating the models: robust comparative fit index (CFI) and robust root mean square error of approximation (RMSEA) test with 90% confidence intervals (CI). CFI values ≥ 0.95 and RMSEA values < 0.05 (Hu & Bentler, 1999) can be considered well accepted model fits.

Our first model estimates handling missing data with the full information maximum likelihood procedure (FIML) did not yield sufficient model fits (CFI, RMSEA) for all four dimensions of value-related educational goals analyzed and the perceived value-related school climate. For this reason, the Multivariate Imputation by Chained Equations (MICE) package (3.16.0) in R was ultimately used to impute missing data in the dataset by filling in missing data not just once, but multiple times. Each “filled in” version is thereby a plausible guess, reflecting the uncertainty about what value to fill in. This process is also known as “chained equations” because the imputation model for each variable is conditional on the imputations for all other variables.

#### Imputation of missing data using MICE

To address the issue of missing data in the dataset, multiple imputation using the *Multivariate Imputation by Chained Equations* (MICE) algorithm was performed. The MICE algorithm is a flexible and robust method for handling missing data, particularly when the data is missing completely at random (MCAR). In this study, the MICE algorithm using the R package “mice” (Buuren and Groothuis-Oudshoorn, 2011) was applied to generate five imputed datasets. The results presented here are the pooled estimates across these datasets using Rubin (1987).

#### Sensitivity analysis for the RI-CLPM

To examine the robustness and generalizability of the RI-CLPMs conducted, we conducted a multi-step sensitivity analysis. First, we reutilized multiple imputation (with 30 imputed datasets) to explore the influence of the imputation process on the results. To assess the sensitivity of our results to the imputation method, models using the initial five imputed datasets were compared with models created using 30 imputed datasets. Results for the *Innovation–Openness to Change* and *Stability–Conservation* model comparison found that most findings (59% and 82% respectively) were largely consistent across the two imputation methods for both models. Notably, this uniformity was evident in the between-level paths and within-level correlations, suggesting these relationships are less sensitive to the number of imputed datasets. A comparison of the within-level correlations, carry-over and spill-over effects revealed that for the *Innovation* (VrSC_Inno) and *Openness to Change* (VrEG_OtC) models, certain paths exhibited divergent outcomes for the two methods. Specifically, in the carry-over effects, the path from VrSC_Inno_t3 to VrSC_Inno_t4 showed a divergence. Additionally, in the spill-over effects, variations were noted in the paths from VrSC_Inno_t1 to VrEG_OtC_t2, from VrEG_OtC_t2 to VrSC_Inno_t3, and from VrEG_OtC_t3 to VrSC_Inno_t4. These differences highlight the sensitivity of these particular paths to the number of imputations, suggesting a need for cautious interpretation in these areas. Similarly, for the Stability (VrSC_Stab) and Conservation (VrEG_Cons) model, distinct differences were also observed, but in different aspects of the model. The carry-over effect from VrSC_Stab_t3 to VrSC_Stab_t4 differed between the models. Within the scope of spill-over effects, divergences appeared in the path from VrSC_Stab_t3 to VrEG_Cons_t4 and from VrEG_Cons_t2 to VrSC_Stab_t3. In summary, while the consistency between different numbers of imputed datasets provides a degree of confidence in the robustness of our models’ outcomes, the identified differences in specific carry-over and spill-over paths highlight areas where interpretations need to be more cautiously approached.

In a second step, the residuals from the primary models (5MI) were inspected to detect potential areas of misfit. The residuals were generally small and randomly distributed, indicating good model fit across observed variables. However, across all imputed datasets in the value-related school climate of *Innovation* (VrSC_Inno) and the value-related educational goals *Openness to Change* (VrEG_OtC) model, the observed covariance between VrEG_OtC_t4 and VrEG_OtC_t2 consistently deviates from what the model predicts. This indicates that there might be some systematic discrepancy between the model and the observed data for this specific relationship. The examination of residuals across imputed datasets for the value-related school climate of *Stability* (VrSC_Stab) and the value-related educational goals *Conservation* (VrEG_Cons) model highlighted four relationships with large residuals. Notably, significant residuals were identified between VrEG_Cons_t1 and VrSC_Stab_t4, VrEG_Cons_t2 and VrSC_Stab_t4, VrEG_Cons_t4 and VrEG_Cons_t2, and between VrSC_Stab_t4 and VrEG_Cons_t2. These findings underscore the need for careful consideration and potential model refinement in these specific areas.

## Results

### Value-related educational goals of openness to change and value-related school climate of innovation

Descriptive analysis for value-related educational goals of *Openness to Change* and the perceived value-related school climate of *Innovation* are reported in Table 1. Moderate significant correlations (*p* < 0.01 and *p* < 0.05 (2-sided)) can be found for all items within the value-related educational goals of *Openness to Change* and the perceived value-related school climate of *Innovation* dimensions. Between the two dimensions, non-significant, low correlations can be reported.

**Table 1:** Descriptive statistics and correlations for value-related school climate of Innovation (VrSC_Inno) and value-related educational goals Openness to Change (VrEG_OtC); t1-t4

Variable	VrSC_Inno_t1	VrSC_Inno_t2	VrSC_Inno_t3	VrSC_Inno_t4	VrEG_OtC_t1	VrEG_OtC_t2	VrEG_OtC_t3	VrEG_OtC_t4
VrSC_Inno_t1	–							
VrSC_Inno_t2	0.417**	–						
VrSC_Inno_t3	0.454**	0.617**	–					
VrSC_Inno_t4	0.472**	0.460**	0.581**	–				
VrEG_OtC_t1	− 0.197*	− 0.051	− 0.171	− 0.150	–			
VrEG_OtC_t2	0.046	0.073	0.175	0.300**	− 0.380**	–		
VrEG_OtC_t3	0.065	0.028	0.155	0.283*	− 0.454**	0.527**	–	
VrEG_OtC_t4	0.071	0.125	0.183	0.294**	− 0.510**	0.548**	0.645**	–
M	0.05	0.00	0.02	0.01	− 0.34	0.22	0.19	0.24
SD	0.44	0.46	0.45	0.53	0.43	0.45	0.42	0.44
N	108	102	96	84	108	102	96	84

*Note*. VrSC_Inno_t1: perceived value-related school climate of *Innovation* at time point *t1.* VrSC_Inno_t2: perceived value-related school climate of *Innovation* at time point *t2.* VrSC_Inno_t3: perceived value-related school climate of *Innovation* at time point *t3.* VrSC_Inno_t4: perceived value-related school climate of *Innovation* at time point *t4.* VrEG_OtC_t1: value-related educational goals *Openness to Change* at time point *t1.* VrEG_OtC_t2: value-related educational goals *Openness to Change* at time point *t2.* VrEG_OtC_t3: value-related educational goals *Openness to Change* at time point *t3.* VrEG_OtC_t4: value-related educational goals *Openness to Change* at time point *t4*

*Correlation statistically significant at *p* < 0.05 (2-sided)

**Correlation statistically significant at *p* < 0.01 (2-sided)

The tested RI-CLPM Model for value-related educational goals *Openness to Change* (VrEG_OtC) and the perceived value-related school climate of *Innovation* (VrSC_Inno) showed a good fit with the data *χ*^2^ (9) = 16.94; *p* > 0.05; CFI = 0.961; RMSEA = 0.086; 90% CI [0.003, 0.148]. In summary, the model appears to fit the data reasonably well according to the chi-square test and CFI. However, the RMSEA and its confidence interval suggest that there might be some aspects of the data that the model does not fit well. The path analysis of this model showed statistically significant *carry-over* (within-subject) effects for VrEG_OtC of 0.644 for *wave_2* (time points *t2-t3*) and of 0.599 for *wave_3* (time points *t3-t4*) and of 0.342 for *wave_2* and 0.366 for *wave_3* for VrSC_Inno. Further statistically significant *spill-over* (within-subject) effects from VrEG_OtC to VrSC_Inno for *wave_2* and *wave_3* and vice versa from VrSC_Inno to VrEG_OtC for *wave_1* (time points *t1-t2*) were also found.

**Fig. 3:**
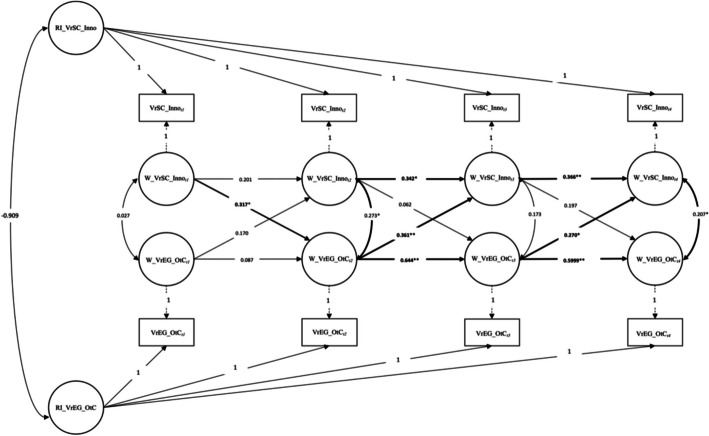
Random Intercept Cross-Lagged Panel Model (RI-CPLPM) for the value-related school climate of Innovation (VrSC_Inno) and value-related educational goals Openness to Change (VrEG_OtC); t1-t4. *Note*. VrSC_Inno_t1: perceived value-related school climate of *Innovation* at time point *t1.* VrSC_Inno_t2: perceived value-related school climate of *Innovation* at time point *t2.* VrSC_Inno_t3: perceived value-related school climate of *Innovation* at time point *t3.* VrSC_Inno_t4: perceived value-related school climate of *Innovation* at time point *t4.* VrEG_OtC_t1: value-related educational goals *Openness to Change* at time point *t1.* VrEG_OtC_t2: value-related educational goals *Openness to Change* at time point *t2.* VrEG_OtC_t3: value-related educational goals *Openness to Change* at time point *t3.* VrEG_OtC_t4: value-related educational goals *Openness to Change* at time point *t4.* *Correlation statistically significant at *p* < 0.05 (2-sided). **Correlation statistically significant at *p* < 0.01 (2-sided)

It can be seen that for the value-related educational goals *Openness to Change* and the perceived value-related school climate of *Innovation* at the beginning of the longitudinal study at *wave_1* (*t1-t2*; March–May 2021 to August-October 2021) the perceived value-related school climate significantly predicted teachers' later value-related educational goals. Values of the school climate perceived by the teachers, for example as: “… we are encouraged to develop ideas of our own” (Item VrSC_Inno_1), “… we are encouraged to look for new ways for doing our jobs.” (VrSC_Inno_2) or “… we are encouraged at my school to look for new ways for solving problems” (VrSC_Inno_3) therefore predicted teachers’ value-related educational goals in the sense of Schwartz’s value types *Hedonism* (pleasure, enjoying live), *Stimulation* (a varied life, an exciting live, daring), and *Self-Direction* (independent thoughts and action-choosing, creating, exploring) (Schwartz, 1992; p. 5–8). The full results are shown in Table 2 and Fig. 3.

**Table 2:** Path coefficients for the Random Intercept Crossed-Lagged Panel Model (RI-CLPM) for the value-related school climate of Innovation (VrSC_Inno) and value-related educational goals Openness to Change (VrEG_OtC); t1-t4

Between-level	*Path*	*Std*	*SE*	*p-value*
VrEG_OtC ↔ VrSC_Inno	j1	-0.909	0.043	0.221
**Within-level**				
*Correlations*				
VrSC_Inno_t1 ↔ VrEG_OtC_t1	j2	0.116	0.050	0.587
VrSC_Inno_t2 ↔ VrEG_OtC_t2	j3	0.273*	**0.025**	**0.040**
VrSC_Inno_t3 ↔ VrEG_OtC_t3	j4	0.173	0.015	0.138
VrSC_Inno_t4 ↔ VrEG_OtC_t4	j5	0.272*	**0.020**	**0.015**
*Carry-over effects*				
VrSC_Inno_t1 → VrSC_Inno_t2	a1	0.201	0.186	0.266
VrSC_Inno_t2 → VrSC_Inno_t3	a2	0.342*	**0.137**	**0.012**
VrSC_Inno_t3 → VrSC_Inno_t4	a3	0.366**	**0.163**	**0.006**
VrEG_OtC_t1 → VrEG_OtC_t2	d1	0.087	0.126	0.546
VrEG_OtC_t2 → VrEG_OtC_t3	d2	0.644**	**0.098**	**0.000**
VrEG_OtC_t3 → VrEG_OtC_t4	d3	0.599**	**0.101**	**0.000**
*Spill-over effects*				
VrSC_Inno_t1 → VrEG_OtC_t2	b1	0.317*	**0.222**	**0.043**
VrSC_Inno_t2 → VrEG_OtC_t3	b2	0.062	0.137	0.540
VrSC_Inno_t3 → VrEG_OtC_t4	b3	0.197	0.138	0.052
VrEG_OtC_t1 → VrSC_Inno_t2	g1	0.170	0.080	0.191
VrEG_OtC_t2 → VrSC_Inno_t3	g2	0.361**	**0.082**	**0.002**
VrEG_OtC_t3 → VrSC_Inno_t4	g3	0.270*	**0.105**	**0.011**

*Note*. The following standardized (Std) parameters and their standard errors (SE) are reported: (1) between-level correlation ( ↔) between the two random intercepts (RI) of VrEG_OtC and VrSC_Inno; (2) within-level correlations ( ↔) between the residual components of VrEG *Openness to Change* (VrEG_OtC) and VrSC *Innovation* (VrSC_Inno) for time points *t1-t4*; (3) within-level *carry-over effects* for VrEG_OtC and VrSC_Inno ( →) for time points *t1-t4*; and (4) within-level *spill-over effects* for VrEG_OtC and VrSC_Inno ( →)

*p value < 0.05

**p value < 0.01

### Value-related educational goals of conservation and value-related school climate of stability

Descriptive analysis for value-related educational goals *Conservation* and the perceived value-related school climate of *Stability* are reported in Table 3. Moderate significant correlations (*p* < 0.01 and *p* < 0.05 (2-sided)) can be found for all items within the value-related educational goals of *Conservation* and the perceived value-related school climate of *Stability* dimensions. Between the two dimensions low, non-significant correlations can be reported.

**Table 3:** Descriptive statistics and correlations for value-related school climate of Stability (VrSC_Stab) and value-related educational goals Conservation (VrEG_Cons); t1-t4

Variable	VrSC_Stab_t1	VrSC_Stab_t2	VrSC_Stab_t3	VrSC_Stab_t4	VrEG_Cons_t1	VrEG_Cons_t2	VrEG_Cons_t3	VrEG_Cons_t4
VrSC_Stab_t1	–							
VrSC_Stab_t2	0.374**	–						
VrSC_Stab_t3	0.447**	0.376**	–					
VrSC_Stab_t4	0.446**	0.380**	0.525**	–				
VrEG_Cons_t1	− 0.193*	− 0.108	0.002	0.314**	–			
VrEG_Cons_t2	0.158	0.129	0.140	0.396**	− 0.369**	–		
VrEG_Cons_t3	0.137	0.022	0.113	0.248*	− 0.577**	0.555**	–	
VrEG_Cons_t4	0.294**	0.044	0.303**	0.471**	− 0.571**	0.637**	0.614**	–
M	− 0.20	− 0.19	− 0.10	− 0.14	0.65	− 0.41	− 0.35	− 0.42
SD	0.49	0.56	0.51	0.56	0.53	0.58	0.48	0.48
N	108	102	96	84	108	102	96	84

*Note*. VrSC_Stab_t1: perceived value-related school climate of *Stability* at time point *t1.* VrSC_Stab_t2: perceived value-related school climate of *Stability* at time point *t2.* VrSC_Stab_t3: perceived value-related school climate of *Stability* at time point *t3.* VrSC_Stab_t4: perceived value-related school climate of *Stability* at time point *t4.* VrEG_Cons_t1: value-related educational goals *Conservation* at time point *t1.* VrEG_Cons_t2: value-related educational goals *Conservation* at time point *t2.* VrEG_Cons_t3: value-related educational goals *Conservation* at time point *t3.* VrEG_Cons_t4: value-related educational goals *Conservation* at time point *t4*

*Correlation statistically significant at *p* < 0.05 (2-sided)

**Correlation statistically significant at *p* < 0.01 (2-sided)

The tested RI-CLPM Model for value-related educational goals *Conservation* (VrEG_Cons) and the perceived value-related school climate of *Stability* (VrSC_Stab) showed a fit with the data *χ*^2^ (9) = 7.716, *p* > 0.05, CFI = 0.937, RMSEA = 0.09; 90% CI [0.000, 0.092].^3^ Overall, the combination of fit indices provided support for the hypothesized conceptual model representing a good fit with the observed data.

Path analysis from this model showed statistically significant *carry-over* (within-subject) effects for VrEG_Cons of 0.712 at *wave_2* (time points *t2-t3*) and 0.617 at *wave_3* (time points *t3-t4*) and of 0.356 for VrSC_Stab at *wave_3*. Further statistically significant *spill-over* (within-subject) effects of 0.258 from VrSC_Stab to VrEG_Cons at *wave_3,* but no vice versa effects from VrEG_Cons to VrSC_Stab over all waves.

**Fig. 4:**
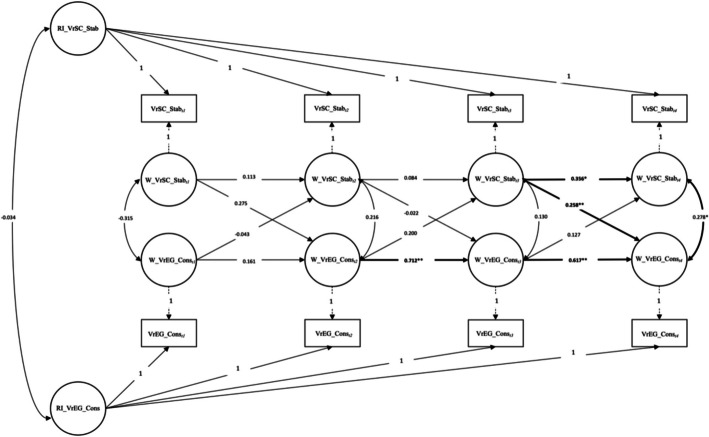
Random Intercept Cross-Lagged Panel Model (RI-CLPM) for value-related school climate of Stability (VrSC_Stab) and value-related educational goals Conservation (VrEG_Cons); t1-t4. *Note*. VrSC_Stab_t1: perceived value-related school climate of *Stability* at time point *t1.* VrSC_Stab_t2: perceived value-related school climate of *Stability* at time point *t2.* VrSC_Stab_t3: perceived value-related school climate of *Stability* at time point *t3.* VrSC_Stab_t4: perceived value-related school climate of *Stability* at time point *t4.* VrEG_Cons_t1: value-related educational goals *Conservation* at time point *t1.* VrEG_Cons_t2: value-related educational goals *Conservation* at time point *t2.* VrEG_Cons_t3: value-related educational goals *Conservation* at time point *t3.* VrEG_Cons_t4: value-related educational goals *Conservation* at time point *t4.* **Correlation statistically significant at *p* < 0.01 (2-sided). *Correlation statistically significant at *p* < 0.05 (2-sided)

Values of the school climate *Stability* were perceived by teachers, for example, as: “… there is a sense of stability and security “ (Item VrSC_Stab_1), “ … high priority is given to working by rules and regulations” (VrSC_Stab_2) or “… high priority is given to being organized and orderly” (VrSC_Stab_3) therefore predicted teachers’ value-related educational goals in the sense of Schwartz’s value types *Tradition* (respect, commitment, and acceptance of the customs and ideas that one’s culture or religion impose on the individual), *Conformity* (restraint of actions, inclinations, and impulses likely to upset or harm others and violate social expectations or norms), and *Security* (safety, harmony, and stability of society, of relationships, and of self) (Schwartz, 1992; p. 10) from time point *t3* to time point *t4*. The full results are shown in Table 4 and Fig. 4, respectively.

**Table 4:** Path coefficients for the Random Intercept Crossed-Lagged Panel Model (RI-CPLM) for value-related school climate of Stability (VrSC_Stab) and value-related educational goals Conservation (VrEG_Cons); t1-t4

Between-level	*Path*	*Std*	*SE*	*p value*
VrEG_Cons ↔ VrSC_Stab	j1	− 0.315	0.039	0.392
**Within-level**				
*Correlations*				
VrSC_Stab_t1 ↔ VrEG_Cons_t1	j2	0.066	0.052	0.673
VrSC_Stab_t2 ↔ VrEG_Cons_t2	j3	0.216	0.039	0.102
VrSC_Stab_t3 ↔ VrEG_Cons_t3	j4	0.130	0.023	0.287
VrSC_Stab_t4 ↔ VrEG_Cons_t4	j5	0.278*	**0.027**	**0.017**
*Carry-over effects*				
VrSC_Stab_t1 → VrSC_Stab_t2	a1	0.113	0.183	0.507
VrSC_Stab_t2 → VrSC_Stab_t3	a2	0.084	0.148	0.571
VrSC_Stab_t3 → VrSC_Stab_t4	a3	0.356**	**0.141**	**0.005**
VrEG_Cons_t1 → VrEG_Cons_t2	d1	0.161	0.127	0.303
VrEG_Cons_t2 → VrEG_Cons_t3	d2	0.712**	**0.094**	**0.000**
VrEG_Cons_t3 → VrEG_Cons_t4	d3	0.617**	**0.097**	**0.000**
*Spill-over effects*				
VrSC_Stab_t1 → VrEG_Cons_t2	b1	0.275	0.258	0.068
VrSC_Stab_t2 → VrEG_Cons_t3	b2	− 0.022	0.119	0.803
VrSC_Stab_t3 → VrEG_Cons_t4	b3	0.258**	**0.127**	**0.007**
VrEG_Cons_t1 → VrSC_Stab_t2	g1	− 0.043	0.067	0.732
VrEG_Cons_t2 → VrSC_Stab_t3	g2	0.200	0.09	0.169
VrEG_Cons_t3 → VrSC_Stab_t4	g3	0.127	0.085	0.177

*Note.* The following standardized (Std) parameters and their standard errors (SE) are reported: (1) between-level correlation ( ↔) between the two random intercepts (RI) of VrEG_Cons and VrSC_Stab; (2) within-level correlations ( ↔) between the residual components of VrEG Conservation (VrEG_Cons) and VrSC Stability (VrSC_Stab) for time points *t1-t4*; (3) within-level *carry-over effects* for VrEG_Cons and VrSC_Stab ( →) for time points *t1-t4*; and (4) within-level *spill-over effects* for VrEG_Cons and VrSC_Stab ( →)

*p value < 0.05

**p value < 0.01

### Value-related educational goals of self-enhancement and value-related school climate of performance and value-related educational goals of self-transcendence and value-related school climate of support

The two theoretically justifiable models for the dimensions of value-related educational goals *Self-Enhancement* (VrEG_SeEn) and value-related school climate of *Performance* (VrSC_Perf) and value-related educational goals *Self-Transcendence* (VrEG_SeTr) and value-related school climate of *Support* (VrSC_Supp) achieved perfect goodness-of-fit with CFI values of 1 and RMSEA values of 0. Evaluating models with a perfect fit requires careful consideration due to several methodological and theoretical caveats. The primary concern was that overfit models would not generalize well to other samples, severely limiting their predictive power and applicability beyond the initial study parameters (Cheung & Rensvold, 2001). Relatedly, the model's complexity likely violates the principle of parsimony and could result in overly complex interpretations that obscure rather than clarify the phenomena under investigation (Barrett, 2007). The need to further investigate the drivers of the aforementioned perfect fit models led to the decision to exclude them from further evaluation. The results for these dimensions are shown in Tables 5 and 6 and in Fig. 5 for VrEG_SeEn and VrSC_Perf and in Tables 7 and 8 and in Fig. 6 for VrEG_SeTr and VrSC_Supp respectively in the Supplementary section.

## Discussion

There is a solid research base on how school stakeholders shape their school environments (c.f., Gálvez-Nieto et al., 2022; Luengo Kanacri et al., 2017; Berson and Oreg, 2016; Haynes et al., 1997), which highlights the role of values within the school culture in shaping school climate. Simultaneously, the school climate encompasses teachers’ perceptions of their work environment including all the unwritten beliefs, values, and attitudes that govern interactions among students, teachers, and the principal (Welsh, 2000).

At present, the authors are unaware of any studies that have previously investigated the interplay between educational goals (i.e., socializing goals) of teachers and their school climate. The present study is the first empirical study to analyze the relationship between teachers’ value-related educational goals and their perceived value-related school climate over time to better understand value transmission in the school context (c.f., Multrus, 2008; Standop, 2013; Stein, 2008). Consequently, our novel study provides new insights into the complex mechanisms of value transmission in schools, thus helping to complement and further develop previous work in this field (c.f., Auer et al., 2023; Barni et al., 2018; Berson & Oreg, 2016; Daniel et al., 2013; Luengo Kanacri et al., 2017; Oeschger et al., 2022; Scholz-Kuhn et al., 2023.)

Our results revealed that teachers’ value-related educational goals and their perceived value-related school climate mutually predict each other over time. Our findings suggest that values in the school environment are not only constituted from a “top-down” approach but also originate from a “bottom-up” approach highlighting the bi-directionality of the value genesis in the school. These findings are in line with Çalık and Kurt (2010), who state that school climate affects the attitudes and behaviors of individuals within school on the one hand, but on the other, consists of various concepts such as atmosphere, culture, character, organizational ideology, climate, values, norms, beliefs, expectations, attitudes, or behaviors of individuals within schools. It can be further assumed that school climate has a multidimensional structure whereby it is simultaneously influenced by the components of the school's leadership but also has a reciprocal effect on them (Ozdemir & Cakalci, 2022).

### Value-related educational goals of openness to change and value-related school climate of innovation

The findings of the current study uncovered reciprocal relationships between teachers' perceptions of their value-related educational goals *Openness to Change* and their perceived value-related school climate of *Innovation*. At the beginning of our longitudinal study in *wave_1* (*t1-t2*; March–May 2021 to August-October 2021), it appeared that teachers’ perceptions of their value-related school climate shaped their value-related educational goals at time point *t2*. One possible reason for this may be that the schools were affected by policy and legally imposed COVID-19 measures at time point *t1*, as pandemic waves 2 and 3 (Oxenius & Karrer, 2022) were taking place in Switzerland at this time. The implementation of the measures had a major influence on the school climate and thus how it was perceived by the teacher. Consequently, this may have led to an influence on the value-related educational goals of the teachers at the later measurement time point *t2* in terms of the *Higher Order Value Type* of *Openness to Change* (*Stimulation*, *Self-Direction* and *Hedonism*). Interestingly, this pattern changed in the opposite direction in the further course of our study. At the subsequent survey waves *wave_2* (*t2-t3*, August–October 2021 to January–February 2022) and survey *wave_3* (*t3-t4*, January–February 2022 to May–June 2022) teachers’ value-related educational goals appeared to influence teachers’ perception of their value-related school climate. It is conspicuous that the direction of the significant influences between teachers’ value-related educational goals and their perceived value-related school climate changed towards the end of the survey. Survey *wave_2* (t2-t3, from August–October 2021 to January–February 2022) and *wave_3* (*t3-t4*, from January–February 2022 to May–June 2022) coincide with a new pandemic situation in Switzerland, when regulations in society and schools were eased again.

Tulviste et al. (2012) reported that guardians differentiate between “short-term” and “long-term” educational goals. Tamm et al. (2020) found that the socialization goals of kindergarten teachers (teaching children up to age 7 years) and secondary school teachers (teaching children up to age 18 years) differ in terms of *Higher Order Value Types* and hypothesized that, “in kindergartens, teachers are likely to be more oriented to short-term socialization values to better prepare children for upcoming school years” (p. 328).

Value-related educational goals of parents (Makarova et al., 2018) as well as teachers are constituted by prevailing social norms, values, and expectations (Veugelers and Vedder, 2003). We therefore assume that the pandemic-related burden on schools at that time—similar to other existential threats such as financial crises (Sortheix et al., 2019), war (Daniel et al., 2013), or terrorist attacks (Verkasalo et al., 2006)—caused a change in teachers’ value-related educational goals at the time of our survey.

This is particularly relevant when it comes to *Conservation* values (the motivation to maintain order and safety, resistance to change) and *Openness to Change* values (the motivation to promote creativity, independence, novelty, and excitement; Daniel et al., 2021). Our findings suggest that teachers had to prepare their students not only for the coming school years (Tamm et al., 2020), but also for changing social conditions after the easing of the pandemic measures. The “pandemic-related shift” in teachers’ value-related educational goals coincides chronologically with the further easing of measures in Swiss society at time point *t2* (August-October 2021). Consequently, due to the significant easing of social measures in society as well as in the school environment, teachers indicated a perception corresponding to their perceived value-related school climate.

### Value-related educational goals of conservation and value-related school climate of stability

School as an institution can generally be regarded as stable. Stakeholders involved in school organization, such as teachers, parents, or pupils, generally have an unconscious basic knowledge of what school structures look like (Tyack & Cuban, 1995). When examining the relationships between teachers’ value-related educational goals of *Conservation* and their perceived value-related school climate of *Stability*, we also found no mutual influence of teachers’ value-related educational goals of *Conservation* and their perceived value-related school climate of *Stability* over the entire measurement period. Only at the end of our survey, at *wave_3* (*t3-t4*, from January–February 2022 to May–June 2022), did the value-related school climate of *Stability* perceived by the teachers predict their value-related educational goals of *Conservation* at the later time point (*t4)*. These findings could be interpreted in light of the major societal event that was deeply affecting the whole world at that time. In January 2022, Russian troops were concentrated along the border of Ukraine and in February 2022 Russia started its full-scale invasion. As a result, European societies in general and schools in particular were confronted with the situation of immediately accommodating refugees from Ukraine. This had a major impact on the school environment in Switzerland. We assume that these sudden drastic structural-systemic changes in the school environment led to teachers’ perception of the value-related school climate influencing their value-related educational goals—in relation to the *Higher Order Value Type* of *Conservation* (with the value types of *Security*, *Stability* and *Tradition*) − shaping their ideas and goals of what they wanted to convey to the children in relation to these values during this time period.

### Limitations

#### Other factors influencing a value-related school climate

Given the non-experimental nature of our research, the possibility of additional factors influencing teachers’ perception of their value-related school climate and their value-related educational goals cannot be ruled out. For example, the location of the school and the values of the guardians of the school’s students (Holme, 2002), the school principals’ values (Hambrick & Mason, 1984), influence a school climate when it comes to the values that prevail therein. It would have been valuable to consider collecting data from additional sources as well in order to obtain an even more comprehensive picture of the genesis and the mutual influence of these components constituting a value-related school climate.

#### Teachers’ age

Butovskaya and Demianovitsch (2002) found that the teacher’s age has an influence on the prioritization of values when it comes to socialization goals. Thus, it would be worth considering the age of the teachers as a control variable when examining the interplay between their value-related educational goals and their perceived school climate, as age seems to be a factor that should not be disregarded.

#### Teacher’s own values and their value-related educational goals

This study focused on value-related educational goals because it is embedded in a larger study investigating key factors on children’s values development in schools. However, there is little empirical research on how the two constructs of personal values and value-related educational goals influence each other and to what context educators in the school context distinguish between these two constructs. Teachers’ value-related educational goals express *explicitly* to what extent teachers want their pupils to endorse values that underlie a social norm, whereas their personal values may be *implicitly* expressed in everyday actions and behaviors and do not necessarily have to coincide with their own values. The values a person holds may differ from the values he or she ideally wants to convey in a targeted manner, e.g., as an educator. In other words, what teachers consider important to their own lives and how they would like to see their students does not necessarily have to be congruent. For the family environment, there are several research findings on the difference between parents’ own values and their value-related educational orientation (c.f., Barni et al., 2017; Makarova et al., 2018; Döring et al., 2017; Benish-Weisman et al., 2013; Knafo & Schwartz, 2009; Rohan & Zanna, 1996; Whitbeck & Gecas, 1988). But to date, there are only limited findings for the school environment, except of findings from Pudelko and Boon (2014) who found that teachers’ classroom goals are affected by teachers’ values and from Tamm et al. (2020) revealing that the socialization goals of teachers can differ from their own values.

#### Methodological aspects

For a longitudinal research design the sample size was relatively small. Although the study operated in a strict methodologically confident manner by using various complex statistical methods, it should be noted that a larger sample would have provided us with additional interesting results, especially for the two missing models with the dimensions of value-related educational goals *Self-Transcendence* versus a value-related school climate of *Support* and value-related educational goals *Self-Enhancement* versus a value-related school climate of *Performance*. Furthermore, a more balanced distribution of the sample in terms of gender would have been desirable to be able to investigate possible gender-related moderation effects, but this was not feasible at the school level investigated due to the given demographic distribution of teachers in Switzerland where 94.6% of the primary school teachers in class 1 and 2 are female (Bundesamt für Statistik (BfS), 2022).

We decided to test four models in our study. One for each *Higher Order Value Type* and each dimension of perceived value-related school climate (see Fig. 1). Schwartz’s *Theory of Basic Human Values* lends itself to this design in that the scales used for the two constructs (dimensions of *Higher-Order Value Types*) are the same and can thus be readily compared statistically (see Fig. 1). This decision was based on the limited sample size and the complexity of the models in our study. The actual design led to a reduction in the number of variables in the model and supported the achievement of robust models. Following Schwartz’s theoretical model assumes that given values are interconnected and put in a system of relationships (Schwartz, 1992, 1994). Analyzing only two models, each including the opposite poles of one value dimension (i.e., *Performance/Support* for the value-related school climate and *Self-Enhancement/Self-Transcendence* for the value-related educational goals and *Innovation/Stability* for the value-related school climate and *Openness to Change/Conservation* for the value-related educational goals respectively) will therefore provide valuable insights into the interaction of the two constructs. Our sensitivity analysis, particularly in the context of handling missing data, underscores the necessity for cautious interpretation of certain findings. This approach aligns with the broader literature, which acknowledges multiple imputation as a robust method for addressing missing data but also highlights the variability in results stemming from the assumptions of different imputation models (Madley-Dowd et al., 2019). While our analysis benefited from this method’s flexibility, the variations observed in specific areas, such as carry-over and spill-over effects, illustrate the potential of different imputation strategies to influence outcomes. Therefore, while our findings contribute valuable insights, the interpretation of areas sensitive to imputation methods should be approached with caution, reflecting the inherent complexities in modelling missing data.

#### Situational aspects

The pandemic situation and the war in the Ukraine had a vast impact on schools worldwide and one can assume that the results in our study were also affected by this special situation. Further research on our research topic to check the replicability of the results without environmental influences like a pandemic or armed conflict would be warranted. Furthermore, a comparison of our results with value-related educational goals of teachers from other school levels (e.g., secondary schools) would be revealing.

## Conclusion and further directions

We investigated the interplay between value-related educational goals and the value-related school climate perceived by teachers. We focused on these two components, as they both play an important part in the context of values education in schools. There are two main conclusions from our study. Firstly, the two elements—teachers’ value-related educational goals and the perceived value-related school climate—do interact with each other over time. The fact that a value-related school climate perceived by teachers is shaped by the value-related educational goals of the teachers involved in it is of practical interest when it comes to understanding and further developing mechanisms of action regarding the genesis of a value-related school climate in terms of the impact of actors involved in the school environment. Secondly, we assume that both elements and their interaction are influenced by deeper social events, such as in our case the pandemic and the war in the Ukraine. This sheds a new light to the responsiveness of values to larger external conditions at the societal level. This is in line with the results of past studies that show that situational influences such as crises lead to a change in the value orientations of individuals (Daniel et al., 2021; Sneddon et al., 2022).

As mentioned in the limitations, we only surveyed teacher’s value-related educational goals and not their own personal values. Further studies should also collect teachers’ personal values and, analogous to the present study, look at how they interact with their perceived value-related school climate over time. Furthermore, it would be appropriate to replicate our study outside of social events with a major impact on schools, especially to analyze in greater depth the mechanisms of values change in educational goals under specific socially driven impacts as in our case. And as reported in the results section, the analyses of the two other possible dimensions of value-related educational goals *Self-Enhancement* and value-related school climate of *Performance* and value-related educational goals *Self-Transcendence* and value-related school climate of *Support* actually achieved perfect goodness-of-fit with CFI values of 1 and RMSEA values of 0. However, overfit models might not generalize well to other samples, which underscores the importance of robustness and theoretical coherence in model selection. We assume that a larger sample size would have revealed more mutual interactions between these constructs.

Further studies would be valuable in order to determine if our findings are replicated outside of social events with a large impact on schools. This would help to analyze the mechanisms of value change in educational goals under certain societal influences, such as those we encountered, in more detail. In addition, it would be interesting to include teachers’ personal values and thus, analogous to the present study, to analyze how these interact with their perceived value-related school climate over time.

## Supplementary Information

Below is the link to the electronic supplementary material.

Supplementary file1 (DOCX 21 KB)

## Footnotes

1 According to Schwartz (1994), the value type *Hedonism* is related both to *Openness to Change* and to *Self-Enhancement* (therefore, the dotted line in Fig. 2, left side.). In our study, we included *Hedonism* in the *Higher Order Value* of *Type Openness to Change* for the value-related educational goals and to the dimension *Innovation* for the perceived value-related school climate. This is in line with most studies in the field of values research (e.g., Auer et al., 2023).

2 VALISE—The Formation of Children’s Values in School: A Study on Value Development Among Primary School Children in Switzerland and the United Kingdom.

3 The unsatisfactory RMSEA values, highlighted above, suggest that both models are far from perfect (Xia & Yang, 2019). However, while sensitivity analysis was carried out to explore the robustness of the current findings, a number of important caveats need to be considered. First, RMSEA should not be considered in isolation and other fit indices should also be considered when evaluating the overall fit of a model. Second, to account for complexity, RMSEA includes a penalty for few degrees of freedom. Consequently, models with few degrees of freedom, such as the ones included in the current study, often have poor RMSEA even when they fit the data well (Kenny et al., 2015). Finally, the cutoff values for goodness-of-fit indices are largely arbitrary and the SEM literature often cautions against overinterpreting cutoffs (see Fan & Sivo, 2007). In line with this, we maintain the position that model fit interpretation should be holistic and consider the broader context of the study and the nuances of the data. In our study, despite the RMSEA values, the parameters of our model were theoretically sound, practically significant, and in line with established literature. Therefore, while we acknowledge the importance of fit indices like RMSEA in model evaluation, we also emphasize the relevance and contribution of our model to the broader research context.
