# Supplementary material for: The interplay between teachers’ value-related educational goals and their value-related school climate over time
Source: Eur J Psychol Educ. 2024 Jun 17;39(4):3633–60. doi: 10.1007/s10212-024-00849-y (PMC11511753; doi:10.1007/s10212-024-00849-y)
Supplement: Supplementary file 1 — Supplementary file1 (DOCX 21 KB) [file 10212_2024_849_MOESM1_ESM.docx]

# Supplementary

**Table 5**

*Descriptive statistics and correlations for value-related school climate of Performance (VrSC_Perf) and value-related educational goals Self-Enhancement (VrEG_SeEn); t1-t4.*

| Variable | VrSC_Perf_t1 | VrSC_Perf _t2 | VrSC_Perf _t3 | VrSC_Perf_t4 | VrEG_SeEn_t1 | VrEG_SeEn_t2 | VrEG_SeEn_t3 | VrEG_SeEn_t4 |
| --- | --- | --- | --- | --- | --- | --- | --- | --- |
| VrSC_Perf_t1 | --- |  |  |  |  |  |  |  |
| VrSC_Perf_t2 | 0.811** | --- |  |  |  |  |  |  |
| VrSC_Perf_t3 | 0.725** | 0.817** | --- |  |  |  |  |  |
| VrSC_Perf_t4 | 0.705** | 0.694** | 0.657** | --- |  |  |  |  |
| VrEG_SeEn_t1 | -0.003 | 0.025 | 0.061 | 0.023 | --- |  |  |  |
| VrEG_SeEn_t2 | 0.130 | 0.110 | 0.208* | 0.129 | -0.263* | --- |  |  |
| VrEG_SeEn_t3 | 0.128 | 0.130 | 0.213* | 0.136 | -0.279** | 0.468** | --- |  |
| VrEG_SeEn_t4 | 0.007 | -0.113 | 0.031 | -0.073 | -0.241* | 0.403** | 0.472** | --- |
| M | -0.22 | -0.29 | -0.26 | -0.24 | 0.94 | -1.04 | -1.02 | -1.05 |
| SD | 0.57 | 0.66 | 0.59 | 0.63 | 0.70 | 0.70 | 0.69 | 0.73 |
| N | 108 | 102 | 96 | 84 | 108 | 102 | 96 | 84 |

*Note*.

VrSC_Perf_t1 Perceived value-related school climate of *Performance* at time point *t1.*

VrSC_Perf_t2 Perceived value-related school climate of *Performance* at time point *t2.*

VrSC_Perf_t3 Perceived value-related school climate of *Performance* at time point *t3.*

VrSC_Perf_t4 Perceived value-related school climate of *Performance* at time point *t4.*

VrEG_SeEn_t1 Value-related educational goals *Self-Enhancement* at time point *t1.*

VrEG_SeEn_t2 Value-related educational goals *Self-Enhancement* at time point *t2.*

VrEG_SeEn_t3 Value-related educational goals *Self-Enhancement* at time point *t3.*

VrEG_SeEn_t4 Value-related educational goals *Self-Enhancement* at time point *t4.*

** Correlation statistically significant at p < 0.01 (2-sided)

* Correlation statistically significant at p < 0.05 (2-sided)

**Table 6**

*Random Intercept Cross-Lagged Panel Model for value-related school climate of Performance (VrSC_Perf) and value-related educational goals Self-Enhancement (VrEG_SeEn); t1-t4.*

|  | |  |  |  |  |
| --- | --- | --- | --- | --- | --- |
| **Between-level** | | ***Path*** | ***Std*** | ***SE*** | ***p-value*** |
| VrEG_SeEn ↔ VrSC_Perf | | ϕ1 | -1.369 | 0.228 | 0.172 |
| **Within-level** |  |  |  |  |  |
| *Correlations* | |  |  |  |  |
| VrSC_Perf _t1 ↔ VrEG_SeEn_t1 | | ϕ2 | 0.463 | 0.241 | 0.178 |
| **VrSC_Perf _t2 ↔ VrEG_SeEn _t2** | | **ϕ3** | **0.421**** | **0.067** | **0.001** |
| **VrSC_Perf _t3 ↔ VrEG_SeEn _t3** | | **ϕ4** | **0.292*** | **0.042** | **0.027** |
| **VrSC_Perf _t4 ↔ VrEG_SeEn _t4** | | **ϕ5** | **0.462**** | **0.069** | **0.002** |
| *Carry-over effects* | |  |  |  |  |
| **VrSC_Perf _t1 → VrSC_Perf_t2** | | **α1** | **0.542**** | **0.164** | **0.000** |
| **VrSC_Perf _t2 → VrSC_Perf_t3** | | **α2** | **0.384*** | **0.136** | **0.013** |
| VrSC_Perf _t3 → VrSC_Perf_t4 | | α3 | 0.222 | 0.233 | 0.249 |
| VrEG_SeEn_t1 → VrEG_SeEn_t2 | | δ1 | 0.224 | 0.144 | 0.096 |
| **VrEG_SeEn_t2 → VrEG_SeEn_t3** | | **δ2** | **0.731**** | **0.101** | **0.000** |
| VrEG_SeEn_t3 → VrEG_SeEn_t4 | | δ3 | **0.546**** | **0.126** | **0.000** |
| *Spill-over effects* | |  |  |  |  |
| **VrSC_Perf_t1 → VrEG_SeEn_t2** | | **β1** | **0.433**** | **0.402** | **0.002** |
| VrSC_Perf_t2 → VrEG_SeEn_t3 | | β2 | 0.091 | 0.279 | 0.398 |
| **VrSC_Perf_t3 → VrEG_SeEn_t4** | | **β3** | **0.280*** | **0.332** | **0.038** |
| VrEG_SeEn_t1 → VrSC_Perf_t2 | | γ1 | 0.128 | 0.044 | 0.23 |
| **VrEG_SeEn_t2 → VrSC_Perf_t3** | | **γ2** | **0.490**** | **0.046** | **0.000** |
| **VrEG_SeEn_t3 → VrSC_Perf_t4** | | **γ3** | **0.365*** | **0.077** | **0.035** |

*Note.*

The following standardized (Std) parameters and their standard errors (SE) are reported:

- Between-level correlation (↔) between the two random intercepts (RI) of VrEG_SeEn and VrSC_Perf

- Within-level correlations (↔) between the residual components of VrEG Self-Enhancement (VrEG_SeEn) and VrSC Performance (VrSC_Perf) for time points *t1-t4*

- Within-level *carry-over effects* for VrEG_SeEn and VrSC_Perf (→) for time points *t1-t4*

- Within-level *spill-over effects* for VrEG_SeEn and VrSC_Perf (→)

* p-value < 0.05

** p-value < 0.01

**Table 7**

*Descriptive statistics and correlations for value-related educational goals Self-Transcendence (VrEG_SeTr) and value-related school climate of Support (VrSC_Supp); t1-t4*

| Variable | VrSC_Supp_t1 | VrSC_ Supp_t2 | VrSC_Supp_t3 | VrSC_Supp_t4 | VrEG_SeTr_t1 | VrEG_SeTr_t2 | VrEG_SeTr_t3 | VrEG_SeTr_t4 |
| --- | --- | --- | --- | --- | --- | --- | --- | --- |
| VrSC_Supp_t1 | --- |  |  |  |  |  |  |  |
| VrSC_Supp_t2 | 0.721** | --- |  |  |  |  |  |  |
| VrSC_Supp_t3 | 0.554** | 0.608** | --- |  |  |  |  |  |
| VrSC_Supp_t4 | 0.531** | 0.592** | 0.683** | --- |  |  |  |  |
| VrEG_SeTr _t1 | -0.049 | -0.094 | -0.080 | -0.101 | --- |  |  |  |
| VrEG_SeTr _t2 | 0.088 | 0.117 | 0.117 | 0.063 | -0.400** | --- |  |  |
| VrEG_SeTr _t3 | 0.151 | 0.210* | 0.299* | 0.115 | -0.563** | 0.526** | --- |  |
| VrEG_SeTr _t4 | 0.127 | 0.070 | 0.199 | -0.032 | -0.396** | 0.405** | 0.640** | --- |
| M | 0.31 | 0.41 | 0.31 | 0.33 | -1.11 | 1.06 | 1.01 | 1.06 |
| SD | 0.45 | 0.48 | 0.49 | 0.49 | 0.65 | 0.69 | 0.59 | 0.56 |
| N | 108 | 102 | 96 | 84 | 108 | 102 | 96 | 84 |

*Note*.

VrSC_Supp_t1 Perceived value-related school climate of *Supportive* at time point *t1.*

VrSC_Supp_t2 Perceived value-related school climate of *Supportive* at time point *t2.*

VrSC_Supp_t3 Perceived value-related school climate of *Supportive* at time point *t3.*

VrSC_Supp_t4 Perceived value-related school climate of *Supportive* at time point *t4.*

VrEG_SeTr_t1 Value-related educational goals *Self-Transcendence* at time point *t1.*

VrEG_SeTr_t2 Value-related educational goals *Self-Transcendence* at time point *t2.*

VrEG_SeTr_t3 Value-related educational goals *Self-Transcendence* at time point *t3.*

VrEG_SeTr_t4 Value-related educational goals *Self-Transcendence* at time point *t4.*

** Correlation statistically significant at p < 0.01 (2-sided)

* Correlation statistically significant at p < 0.05 (2-sided)

**Table 8**

*Random Intercept Cross-Lagged Panel Model for value-related school climate of Support (VrSC_Supp) and value-related educational goals Self-Transcendence (VrEG_SeTr); t1-t4.*

|  | |  |  |  |  |
| --- | --- | --- | --- | --- | --- |
| **Between-level** | | ***Path*** | ***Std*** | ***SE*** | ***p-value*** |
| VrEG_ *SeTr* ↔ VrSC_Supp | | ϕ1 | -6.089 | 0.174 | 0.240 |
| **Within-level** |  |  |  |  |  |
| *Correlations* | |  |  |  |  |
| VrSC_Supp _t1 ↔ VrEG_*SeTr* _t1 | | ϕ2 | 0.393 | 0.174 | 0.240 |
| VrSC_Supp _t2 ↔ VrEG_*SeTr* _t2 | | ϕ3 | **0.464**** | **0.042** | **0.000** |
| VrSC_Supp _t3 ↔ VrEG_*SeTr* _t3 | | ϕ4 | **0.457**** | **0.031** | **0.000** |
| VrSC_Supp _t4 ↔ VrEG_*SeTr* _t4 | | ϕ5 | **0.439**** | **0.035** | **0.002** |
| *Carry-over effects* | |  |  |  |  |
| **VrSC_Supp _t1 → VrSC_Supp_t2** | | **α1** | **0.576**** | **0.148** | **0.000** |
| VrSC_Supp _t2 → VrSC_Supp_t3 | | α2 | **0.418**** | **0.149** | **0.003** |
| VrSC_Supp _t3 → VrSC_Supp_t4 | | α3 | **0.454**** | **0.149** | **0.004** |
| VrEG_*SeTr* _t1→ VrEG_ *SeTr* _t2 | | δ1 | 0.139 | 0.125 | 0.316 |
| **VrEG_*SeTr* _t2 → VrEG_*SeTr* _t3** | | **δ2** | **0.568**** | **0.108** | **0.000** |
| VrEG_*SeTr* _t3 → VrEG_*SeTr* _t4 | | δ3 | **0.551**** | **0.136** | **0.000** |
| *Spill-over effects* | |  |  |  |  |
| **VrSC_Supp _t1 → VrEG_*SeTr* _t2** | | **β1** | **0.397*** | **0.374** | **0.010** |
| **VrSC_Supp _t2 → VrEG_*SeTr* _t3** | | **β2** | **0.271*** | **0.241** | **0.011** |
| **VrSC_Supp _t3 → VrEG_*SeTr* _t4** | | **β3** | **0.241*** | **0.265** | **0.042** |
| VrEG_*SeTr* _t1 → VrSC_Supp _t2 | | γ1 | 0.041 | 0.040 | 0.692 |
| **VrEG_*SeTr* _t2 → VrSC_Supp _t3** | | **γ2** | **0.308**** | **0.054** | **0.009** |
| VrEG_*SeTr* _t3 → VrSC_Supp _t4 | | γ3 | 0.245 | 0.069 | 0.082 |

*Note.*

The following standardized (Std) parameters and their standard errors (SE) are reported:

- Between-level correlation (↔) between the two random intercepts (RI) of VrEG_SeTr and VrSC_Supp

- Within-level correlations (↔) between the residual components of VrEG Self-Transcencene (VrEG_SeTr) and VrSC Supportive (VrSC_Supp) for time points *t1-t4*

- Within-level *carry-over effects* for VrEG_SeTr and VrSC_Supp (→) for time points *t1-t4*

- Within-level *spill-over effects* for VrEG_SeTr and VrSC_Supp (→)

* p-value < 0.05

** p-value < 0.01
